# Is Being a Boy and Feeling Fat a Barrier for Physical Activity? The Association between Body Image, Gender and Physical Activity among Adolescents

**DOI:** 10.3390/ijerph111111167

**Published:** 2014-10-27

**Authors:** Jaroslava Kopcakova, Zuzana Dankulincova Veselska, Andrea Madarasova Geckova, Jitse P. van Dijk, Sijmen A. Reijneveld

**Affiliations:** 1 Department of Health Psychology, Institute of Public Health, Medical Faculty, Safarik University, Tr. SNP 1, 040 11 Kosice, Slovak Republic; E-Mails: zuzana.veselska@upjs.sk (Z.D.V.); geckova.andrea.madarasova@upjs.sk (A.M.G.); 2 Center for Kinanthropology Research, Institute of Active Lifestyle, Faculty of Physical Culture, Palacky University in Olomouc, Tr. Miru 115, Olomouc 77111, Czech Republic; 3 Olomouc University Social Health Institute (OUSHI), Palacky University in Olomouc, Tr. Miru 115, Olomouc 77111, Czech Republic; E-Mail: j.p.van.dijk@umcg.nl; 4 Department of Community & Occupational Health, University Medical Center Groningen, University of Groningen, A. Deusinglaan 1, 9713 AV Groningen, The Netherlands; E-Mail: s.a.reijneveld@umcg.nl

**Keywords:** adolescents, body image, physical activity, gender

## Abstract

Regular physical activity leads to physical and mental health benefits. Previous studies have shown physical activity to be associated with body image and gender. The aim of this cross-sectional study was to explore the associations of body image with physical activity of adolescents and whether gender modifies this association. We obtained data on body image and physical activity as part of the Health Behaviour in School-Aged Children study in 2010 from Slovakia (*n* = 8042, age 11–15 years, 49% boys, response rate: 79.5%). Adolescents answered questions about their body image and the frequency of their physical activity. Sufficient physical activity was more likely in adolescents perceiving themselves as fat (OR = 0.63, 95%CI 0.54–0.73) and in boys (OR = 2.15, 95%CI 1.92–2.42). A poor body image among girls was not associated with physical activity, whereas among boys it was associated with less physical activity. Gender seems to moderate the relationship between body image and physical activity in youths. Health promotion should be targeted in particular at boys with a negative body image, as they are at higher risk of physical inactivity.

## 1. Introduction

Physical activity is an essential part of a healthy lifestyle in adolescence [1]. Regular physical activity leads to physical and mental health benefits, which can make an important contribution to improving physical and psychological quality of life [2]. Low levels of physical activity during adolescence contribute to obesity and poor health outcomes in adulthood [2,3], and this association endures into adulthood [4]. Physical activity is linked with a number of positive physical and psychological health outcomes [1,3,5]. Generally, findings on physical activity in young people reveal that boys are more active than girls, and that the amount of physical activity decreases with age [4,6,7,8,9,10,11,12].

Body image is a multidimensional construct with attitudinal, perceptual and also behavioural components [13] covering various attributes like muscularity, leanness and body weight. The present study pays special attention to dissatisfaction with body weight as a component of body image, as it has particular importance due to its association with subjective well-being [13,14] and weight-control behaviour, which may manifest itself in both unhealthy (e.g., fasting, purging, smoking, extreme diets or training) and healthy (e.g., healthy diet, appropriate physical activity) lifestyles [7]. Body weight satisfaction may change remarkably during adolescence (especially in puberty) due to rapid and significant somatic changes, and may then have impact on mental well-being and behaviour [15]. Most of the available evidence shows that a more developed pubertal status is associated with a less positive body image, increased body dissatisfaction and increased internalization of thin ideals [6,7,9]. Dissatisfaction with body weight intensifies across adolescence among girls [16] while remaining constant among boys [15]. Dissatisfaction with body weight seems to be associated with a negative body image [15], and gender might modify its effect. International Health Behaviour in School-Aged Children study (HBSC) data on body image have shown consistent patterns with other studies [6,7,16,17,18] that girls have a significantly higher prevalence in perceiving their body as being too fat compared with boys. On the other hand, the pathway to boys’ body dissatisfaction might go through an internalised commitment to muscularity [19] and might be related to both underweight and overweight/obese. According to the findings of Currie *et al.* [16], boys and girls in Western and Central Europe are more likely to report being “too fat” than boys and girls in Eastern Europe.

Several studies have shown that regular physical activity has a beneficial effect on body image perception among children and adolescents. Body image dissatisfaction may be the reason for choosing physical activity and exercise as a strategy for obtaining the optimal image, especially in adolescent girls [20,21], and inversely, body image dissatisfaction was also related with less engagement in physical activities [22]. Regardless, the association between body image dissatisfaction and physical activity has not yet been properly quantified, as previous research was carried out only among specific gender or age subgroups [23,24,25].

Gender seems to play an important role in the connection between body image and physical activity. The association between gender and physical activity was explored in the above-mentioned studies [4,5,6,7,9], and many other studies have unequivocally documented differences in girls’ body image dissatisfaction to be associated with physical activity [26,27,28]. According to our knowledge, the possible effect of boys’ body image dissatisfaction associated with physical activity has also not yet been studied, and this is also of foremost interest. Through this study, we want to explore and clarify the associations of body image and gender with physical activity.

The aim of this study was to explore the associations of body image with physical activity controlled for age, and whether this association is modified by gender crude and after additional adjustment for BMI.

## 2. Methods

### 2.1. Sample and Procedure

We used data from the Health Behaviour in School-aged Children (HBSC) cross-sectional study conducted in 2010 in Slovakia. From a list of schools based on information from the Slovak Institute of Information and Prognosis for Education, 134 larger and smaller schools located in rural as well as in urban areas from all regions of Slovakia were randomly chosen to create a representative sample. We contacted 108 schools, and 106 schools took part in our survey, representing a 98.1% school response rate. According to the protocol of the HBSC study, classes from the 5th–9th grades were selected randomly, one from each grade per school. We obtained data from 8491 adolescents 10–19 years of age (mean age 13.12 years, 48.7% boys) from the 5th–9th grade of elementary schools in Slovakia (response rate 79.5%). Non-response was primarily due to illness (10.3%) and parental disapproval of the participation of their children (7.4%). We decided to exclude children under age 11 and over 15 to make the sample more homogeneous and to avoid the influence of age extremes. After this step, the study sample consisted of 8042 adolescents (mean age 13.13 years, 48.6% boys) from elementary schools in Slovakia.

The study was approved by the Ethics Committee of the Faculty of Medicine at the Safarik University in Kosice. Parents were informed about the study via the school administration and could opt out if they disagreed with participation. Participation in the study was fully voluntary and anonymous with no explicit incentives provided for participation. Questionnaires were administrated by trained research assistants in the absence of a teacher during regular class time.

### 2.2. Measures

Demographic data (age, gender) were collected using questions used and validated in the Health Behaviour in School-Aged Children (HBSC) surveys [6,7,29].

Body image was assessed using the single-item HBSC question asking “Do you think your body is?” with five possible answers ranging from “much too fat” to “much too thin” [6,7]. We dichotomised the answers into two categories—those who felt fat (answers “a bit too fat” and “much too fat”) and those who felt not fat (answers “much too thin”, “a bit too thin” and “about the right size”).

Physical activity was assessed by the single-item HBSC question asking “Over the past 7 days, on how many days were you physically active for a total of at least 60 min per day?” with answers ranging from 0–7 days [6,7]. This item was developed by Prochaska *et al.* [30] to produce a reliable and valid screening measure of moderate to vigorous physical activity of children and adolescents. To assure that respondents will consider the whole variety of physical activity and will take into account intensity, the item is associated with the following introductory instruction: “Physical activity is any activity that increases your heart rate and makes you get out of breath some of the time. Physical activity can be done in sports, school activities, playing with friends, or walking to school. Some examples of physical activity are running, brisk walking, rollerblading, biking, dancing, skateboarding, swimming, soccer, basketball or skiing.” The responses to this question were dichotomised for logistic regression into two categories, with the cut-off point at 7 days of physical activity, further denoted as sufficient (7 days) *vs.* not sufficient physical activity (0–6 days) [3].

Body Mass Index was calculated from the item HBSC questions asking “How much do you weigh with no clothes on?” and “How tall are you with no clothes on?” [6,7]. The responses to this question were used as a continuous variable.

### 2.3. Statistical Analyses

Standard descriptive analyses for the whole study sample as well as for genders were performed in the first step. Next, we explored the prevalence of age, physical activity, body image and body mass index by gender and examined gender differences using chi-square tests to determine statistical significance. In the third step, we used binary logistic regression models adjusted for age to explore the associations of body image with sufficient physical activity, leading to odds ratios (OR) and 95% confidence intervals (CI). In Model 1, we explored the association of body image and gender with physical activity. In Model 2, the interaction between body image and gender was added in order to assess the moderating effect of gender on the association between body image and physical activity. In Model 3, we repeated the analyses and in addition to age we adjusted the analyses also for BMI status. All analyses were performed using the Predictive Analytics Software, Version 18.0 (PASW, Chicago, IL, USA).

## 3. Results

The background characteristics of the sample are present in Table 1, overall and by gender. Statistically significant gender differences were found for all studied variables. Boys reported more physical activity on 7 days/week, higher satisfaction with their body image and higher BMI compared with girls.

Table 2 presents the odds ratios (OR) and 95% confidence intervals (CI) from the logistic regression analyses. In Model 1, significant associations were found between body image and physical activity as well as between gender and physical activity. In Model 2, we found a significant interaction between body image and gender. This showed that poor body image did not affect the physical activity of girls, whereas poor body image was associated with lower probability to reach recommended level of moderate to vigorous physical activity of boys. Next, in Model 3 we adjusted for age and BMI status. This did not change the association between the explored variables to a substantial degree.

**Table 1:** Descriptive statistics for age, physical activity, body image and body mass index, for the whole sample and separately for boys and girls.

	Whole Sample (*n* = 8042)	Boys (*n* = 3910)	Girls (*n* = 4132)	*p*
Age: Mean (SD)	13.13 (1.35)	13.16 (1.35)	13.11 (1.35)	˂0.001 ^a ^
Physical activity: *n* (%)				˂0.001 ^b ^
sufficient	1765 (22.5)	1116 (29.3)	649 (16.1)	
not sufficient	6089 (77.5)	2696 (70.7)	3393 (83.9)	
Body image: *n* (%)				˂0.001 ^b^
fat	1901 (23.9)	766 (19.9)	1135 (27.6)	
not fat	6059 (76.1)	3083 (80.1)	2976 (72.4)	
BMI: Mean (SD)	19.34 (3.19)	19.75 (3.33)	18.96 (2.99)	˂0.001 ^a^

Notes: Number of missing cases per variable: Age—0; physical activity—188; body image—82; BMI—822; BMI, Body mass index; SD, standard deviation; **^a^** *t*-test; **^b^** Chi-square test.

**Table 2:** Associations of body image and gender with sufficient physical activity: Odds ratios (OR) and 95% confidence intervals (95% CI) from binary logistic regression adjusted for age and BMI status.

	Model 1	Model 2	Model 3
	OR (95%CI) Adjusted for Age	OR (95%CI) Adjusted for Age	OR (95%CI) Adjusted for Age and BMI Status
Body image			
not fat (Ref.)	1.00	1.00	1.00
fat	0.63 (0.54–0.73) ***	0.87 (0.71–1.07)	0.97 (0.78–1.20)
Gender			
girls (Ref.)	1.00	1.00	1.00
boys	2.15 (1.92–2.42) ***	2.44 (2.14–2.78) ***	2.50 (2.20–2.86) ***
Body image (fat) × gender (male)		0.53 (0.39–0.71) ***	0.55 (0.41–0.74) ***

Notes: *** *p* < 0.001; Ref. = reference group.

## 4. Discussion

This study explored the associations of body image and gender with physical activity and the potential influence of gender on the association between body image and physical activity of adolescents. The results show that adolescents with a negative body image engage in regular sufficient physical activity less often than others, and that boys are more likely to report sufficient physical activity. Poor body image among girls did not affect their physical activity, whereas poor body image among boys was associated with lower probability to reach the recommended level of moderate to vigorous physical activity.

We found that boys, not girls, are prone to be physically inactive when they are dissatisfied with their body image. This gender difference in the influence of body image seems to originate from different sociocultural expectations. A study of Currie *et al.* [16] reported that boys and girls in Western and Central Europe are more likely to report being “too fat” compared with boys and girls in Eastern Europe. For example, Zach *et al.* [31] reported in their study that the highest percentage of active boys occurred among overweight boys and those who perceived themselves as fat. This is not in line with our findings, probably due to cultural differences. As adolescents are often dissatisfied with their body development, they tend to engage in methods to change their bodies. In general, boys want to increase their muscle mass and tone, and to decrease their fat mass and mainly choose physical activity to do so, while girls want to lose weight and mainly choose dieting or other eating-related methods to change their weight [25]. One possible explanation of the differences in perception of body image could be that influence on body image is transmitted mostly by media images [32], a powerful conduit for the transmission and reinforcement of cultural beliefs and values, although it may not be exclusively responsible for determining the standards for physical attractiveness. Young people are especially responsive to media messages that display perfect and ideal body shapes and are at risk of preoccupation with their physical appearance and developing a negative body image. Stanford and McCabe [25] reported in their study that parents appear to provide the strongest and most consistent messages to young adolescent boys regarding body image.

The association between body image dissatisfaction and physical activity has not yet been properly explored in quantitative research, and there is little evidence on the association between body image and physical activity among boys. Gender seems to play an important role in the connection between body image and physical activity. Gillison *et al.* [28] reported in their study that those adolescent girls who are engaged in physical activity perceive it as a sort of duty, and their motivation is related to their physical attractiveness, health benefits and feeling good about themselves, which may be connected with the way they perceive themselves. Veselska *et al.* [33] reported that in boys self-perception did not play such an important role as in girls, because their motivation for physical activity differed from girls. Boys are more engaged in group sporting activities with the aim of being part of peer relationships, which is not connected directly with the way they perceive themselves. Therefore, in future research it will be also important to take a closer look at the motivation for physical activity and possible barriers to it among adolescents, particularly among adolescent boys.

This study has several important strengths, the most important being the large and representative sample size of adolescents and the high response rate. The main limitation of our study could be that we used subjective self-reports for measuring body image, physical activity and BMI. Anonymity, confidentiality and also privacy were provided by self-administration of questionnaires in the absence of teachers; this decreased the probability of the over- or under-reporting of health-related behavior [34]. Moreover, the questions on physical activity that we used have been shown to have a high validity and reliability [34]. While self-reported data on psychological complaints are a rather preferred source of information, the validity and reliability of self-reported as well as measured PA or sedentary behaviour indicators are discussed heavily in literature [35,36,37,38,39,40]. Neither the self-report data nor the measured data like using accelerometer or pedometer is a gold standard for measuring physical activity, and validation studies are needed to estimate potential bias. A limitation is the cross-sectional design of our study, which makes it impossible to formulate conclusive statements about causality in our findings. Our findings therefore need to be confirmed in studies with a longitudinal design.

Based on our findings, it seems that successful promotion programmes may need to consider gender-specific strategies aiming at girls and boys separately. Adolescent boys with a negative body image are less physically active than other boys, and therefore it could be important to pay attention to this.

Future studies should also further explore the negative body image among boys and not just among girls because recently attention has mostly been paid to the association between body image dissatisfaction and physical activity among girls. Future research should preferably have a longitudinal design in order to be able to assess the causal relationship between physical activity and body image by gender.

## 5. Conclusions

Physical activity is a possible way of enhancing health during adolescence, and the amount of physical activity is not only gender dependent, but also dependent on body image. Adolescent boys with a negative body image are less physically active than other boys. A challenge in health promotion is to maintain their relatively good perception of body image while promoting physical activity. Prevention programmes should target youth by highlighting and promoting a healthy lifestyle also for adolescent boys.
